# (*E*)-Methyl 2-benzyl-3-*o*-tolyl­acrylate

**DOI:** 10.1107/S1600536812013438

**Published:** 2012-04-04

**Authors:** S. Karthikeyan, K. Sethusankar, Anthonisamy Devaraj, Manickam Bakthadoss

**Affiliations:** a Department of Physics, RKM Vivekananda College (Autonomous), Chennai 600 004, India; b Department of Organic Chemistry, University of Madras, Maraimalai Campus, Chennai 600 025, India

## Abstract

In the title compound, C_18_H_18_O_2_, the methyl acrylate substituent adopts an extended *E* conformation with all torsion angles close to 180°. The mean plane of the acrylate unit and the phenyl ring are approximately orthogonal to each other, making a dihedral angle of 81.40 (6)°. The position of the carbonyl group with respect to the olefinic double bond is typically *S*-*trans*. The crystal packing is stabilized by inter­molecular C—H⋯π inter­actions.

## Related literature

For applications of acrylate derivatives, see: Xiao *et al.* (2008 ▶); De Fraine & Martin, (1991 ▶). For a related structure, see: Madhanraj *et al.* (2011 ▶). For *E*-conformation aspects, see: Dunitz & Schweizer (1982 ▶). For resonance effects in acrylate, see: Merlino (1971 ▶); Varghese *et al.* (1986 ▶).
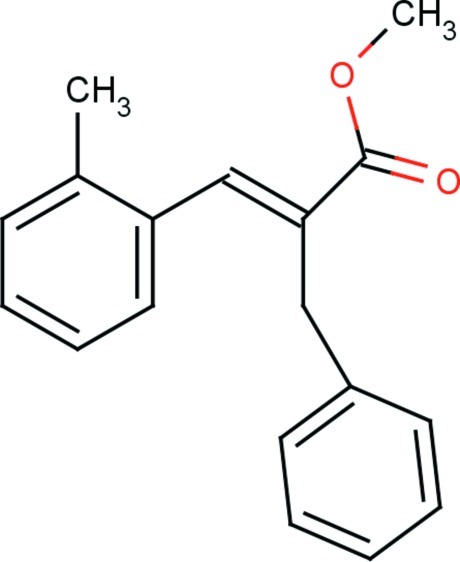


## Experimental

### 

#### Crystal data

C_18_H_18_O_2_
*M* *_r_* = 266.32
Monoclinic, 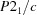

*a* = 7.6277 (3) Å
*b* = 16.2167 (7) Å
*c* = 11.7990 (5) Å
β = 92.419 (2)°
*V* = 1458.19 (11) Å^3^
*Z* = 4
Mo *K*α radiation
μ = 0.08 mm^−1^
*T* = 295 K
0.28 × 0.25 × 0.23 mm

#### Data collection

Bruker Kappa APEXII CCD diffractometer
16396 measured reflections
3397 independent reflections
2183 reflections with *I* > 2σ(*I*)
*R*_int_ = 0.033

#### Refinement

*R*[*F* ^2^ > 2σ(*F* ^2^)] = 0.045
*wR*(*F* ^2^) = 0.127
*S* = 1.03
3397 reflections
183 parameters
H-atom parameters constrained
Δρ_max_ = 0.12 e Å^−3^
Δρ_min_ = −0.20 e Å^−3^

### 

Data collection: *APEX2* (Bruker, 2008 ▶); cell refinement: *SAINT* (Bruker, 2008 ▶); data reduction: *SAINT*; program(s) used to solve structure: *SHELXS97* (Sheldrick, 2008 ▶); program(s) used to refine structure: *SHELXL97* (Sheldrick, 2008 ▶); molecular graphics: *ORTEP-3* (Farrugia, 1997 ▶); software used to prepare material for publication: *SHELXL97* and *PLATON* (Spek, 2009 ▶).

## Supplementary Material

Crystal structure: contains datablock(s) global, I. DOI: 10.1107/S1600536812013438/rk2348sup1.cif

Structure factors: contains datablock(s) I. DOI: 10.1107/S1600536812013438/rk2348Isup2.hkl

Supplementary material file. DOI: 10.1107/S1600536812013438/rk2348Isup3.cml

Additional supplementary materials:  crystallographic information; 3D view; checkCIF report

## Figures and Tables

**Table 1:** Hydrogen-bond geometry (Å, °) *Cg*1 is the centroid of the C13–C18 ring.

*D*—H⋯*A*	*D*—H	H⋯*A*	*D*⋯*A*	*D*—H⋯*A*
C3—H3⋯*Cg*1^i^	0.93	2.87	3.7809 (17)	165

Symmetry code: (i) 
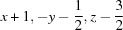
.

**Fig. 1.**
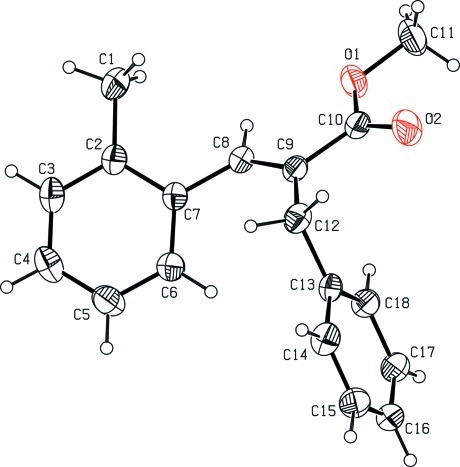
The molecular structure of the title compound with the atom numbering scheme. Displacement ellipsoids are drawn at 30% probability level. H atoms are presented as a small spheres of arbitary radius.

**Fig. 2.**
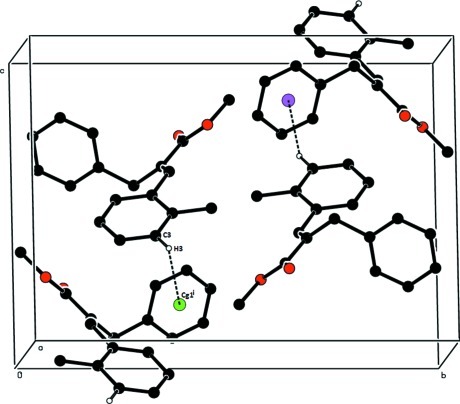
The packing arrangement of the title compound viewed down c axis. The dashed line indicate C3–H3···Cg1i intermolecular interaction. Cg1 is the centroid of the benzene ring (C13-C18). Symmetry code: (i) x+1, -y-1/2, z-3/2.

## supplementary crystallographic information

### Comment

Phenyl acrylate and its derivatives are important compounds because of their agrochemical and medicinal applications (De Fraine & Martin, 1991). Phenyl acrylates show considerable antibacterial activities against *Staphylococcus Aureus* (Xiao *et al.*, 2008).

In the title compound C_18_H_18_O_2_, the methyl acrylate is essentially planar with a maximum deviation of -0.0207 (14)Å for the C9 atom and forms a dihedral angle of 40.66 (6)° and 81.40 (6)° with two phenyl rings (C2-C7) and (C13-C18), respectively. The interplanar angle between the two phenyl rings (C2-C7) and (C13-C18) is 67.69 (7)°. The title molecule exhibits structural similarities with the already reported related structure (Madhanraj *et al.*, 2011).

The significant difference in the length of the C10–O1 = 1.3307 (19) Å and C11–O1 = 1.4368 (18)Å bond is attributed to a partial contribution from O^-^–C═O^+^–C resonance structure of the O2═C10–O1–C11 group (Merlino, 1971). This feature, commonly observed in the carboxylic ester group of the substituents in various compounds gives average values of 1.340Å and 1.447Å respectively for these bonds (Varghese *et al.*, 1986).

The configuration of the keto-group with respect to the olefinic double bond is typically *S*-*trans*, with O2═C10–C9═C8 torsion angle 176.88 (16)°. The methyl acrylate adopts an extended *E*-configuration with the torsion angles C8═C9–C10═O2 = 176.88 (16)°, C8═C9–C10–O1 = -2.2 (2)°, C9–C10–O1–C11 = 179.81 (14)° and C12–C9–C10–O1 = -179.66 (12)°. The extended conformation is supported by the fact that the bond angles involving carbonyl O atoms are invariably expanded (Dunitz & Schweizer, 1982).

The crystal packing is stabilized by intermolecular C–H···π interaction, between a methyl benzene H atom and the benzene ring (C13-C18) of an adjacent molecule, with a C3–H3···*Cg*1^i^ seperation of 2.87Å. *Cg*1 is the centroid of the benzene ring (C13-C18). Symmetry code: (i) *x*+1, -*y*-1/2, *z*-3/2.

### Experimental

To a stirred solution of methyl 2-(hydroxy(*o*-tolyl)methyl)acrylate (0.21 g, 1 mmol) in dichloromethane (10 mL), benzene (0.31 g, 4 mmol) was added at room temperature. After stirring for about 10 minutes at 273 K, catalytic amount of concentrated H_2_SO_4_ was added drop wise. Then the reaction mixture was stirred at room temperature for 6 h. After completion of reaction, the mixture was poured into water and aqueous layer was extracted with ethyl acetate (3×10 ml). The combined organic layer was washed with brine (20 mL), and dried over anhydrous Na_2_SO_4_. The crude product thus obtained was purified by column chromatography (2% *Et*O*Ac* / hexanes) to provide the desired compound (*E*)-methyl-2-benzyl-3-*o*-tolyl acrylate in 80% yield, as a colourless solid.

### Refinement

All the hydrogen atoms of the compound are fixed geometrically and allowed to ride on their parent atoms with C–H distance in the range 0.93Å to 0.97Å and with *U*_iso_(H) = 1.5*U*_eq_(C) for CH_3_ groups and *U*_iso_(H) = 1.2*U*_eq_(C) for all other groups.

### Crystal data

**Table:**

C_18_H_18_O_2_	*F*(000) = 568
*M**_r_* = 266.32	*D*_x_ = 1.213 Mg m^−^^3^
Monoclinic, *P*2_1_/*c*	Mo *K*α radiation, λ = 0.71073 Å
Hall symbol: -P 2ybc	Cell parameters from 3397 reflections
*a* = 7.6277 (3) Å	θ = 2.1–27.7°
*b* = 16.2167 (7) Å	µ = 0.08 mm^−^^1^
*c* = 11.7990 (5) Å	*T* = 295 K
β = 92.419 (2)°	Block, colourless
*V* = 1458.19 (11) Å^3^	0.28 × 0.25 × 0.23 mm
*Z* = 4	

### Data collection

**Table:**

Bruker Kappa APEXII CCD diffractometer	2183 reflections with *I* > 2σ(*I*)
Radiation source: fine-focus sealed tube	*R*_int_ = 0.033
Graphite monochromator	θ_max_ = 27.7°, θ_min_ = 2.1°
ω and φ scans	*h* = −6→9
16396 measured reflections	*k* = −21→20
3397 independent reflections	*l* = −15→15

### Refinement

**Table:**

Refinement on *F*^2^	Primary atom site location: structure-invariant direct methods
Least-squares matrix: full	Secondary atom site location: difference Fourier map
*R*[*F*^2^ > 2σ(*F*^2^)] = 0.045	Hydrogen site location: inferred from neighbouring sites
*wR*(*F*^2^) = 0.127	H-atom parameters constrained
*S* = 1.03	*w* = 1/[σ^2^(*F*_o_^2^) + (0.0583*P*)^2^ + 0.1449*P*] where *P* = (*F*_o_^2^ + 2*F*_c_^2^)/3
3397 reflections	(Δ/σ)_max_ < 0.001
183 parameters	Δρ_max_ = 0.12 e Å^−^^3^
0 restraints	Δρ_min_ = −0.20 e Å^−^^3^

### Special details

**Table:**

Geometry. All s.u.'s (except the s.u. in the dihedral angle between two l.s. planes) are estimated using the full covariance matrix. The cell s.u.'s are taken into account individually in the estimation of s.u.'s in distances, angles and torsion angles; correlations between s.u.'s in cell parameters are only used when they are defined by crystal symmetry. An approximate (isotropic) treatment of cell s.u.'s is used for estimating s.u.'s involving l.s. planes.
Refinement. Refinement of *F*^2^ against ALL reflections. The weighted *R*-factor *wR* and goodness of fit *S* are based on *F*^2^, conventional *R*-factors *R* are based on *F*, with *F* set to zero for negative *F*^2^. The threshold expression of *F*^2^ > σ(*F*^2^) is used only for calculating *R*-factors(gt) *etc*. and is not relevant to the choice of reflections for refinement. *R*-factors based on *F*^2^ are statistically about twice as large as those based on *F*, and *R*-factors based on ALL data will be even larger.

### Fractional atomic coordinates and isotropic or equivalent isotropic displacement parameters (Å^2^)

**Table:**

	*x*	*y*	*z*	*U*_iso_*/*U*_eq_	
C1	0.7834 (3)	0.43187 (11)	0.45429 (16)	0.0774 (6)	
H1A	0.6742	0.4614	0.4486	0.116*	
H1B	0.8577	0.4500	0.3956	0.116*	
H1C	0.8402	0.4422	0.5270	0.116*	
C2	0.74883 (18)	0.34125 (10)	0.44130 (12)	0.0506 (4)	
C3	0.8329 (2)	0.29632 (11)	0.35940 (14)	0.0621 (4)	
H3	0.9075	0.3235	0.3115	0.075*	
C4	0.8091 (2)	0.21326 (12)	0.34737 (15)	0.0675 (5)	
H4	0.8673	0.1846	0.2919	0.081*	
C5	0.6995 (2)	0.17216 (11)	0.41701 (15)	0.0682 (5)	
H5	0.6844	0.1155	0.4098	0.082*	
C6	0.6119 (2)	0.21510 (10)	0.49768 (14)	0.0578 (4)	
H6	0.5372	0.1869	0.5445	0.069*	
C7	0.63261 (17)	0.29962 (9)	0.51076 (11)	0.0456 (3)	
C8	0.54009 (18)	0.34614 (9)	0.59692 (12)	0.0473 (3)	
H8	0.6046	0.3874	0.6342	0.057*	
C9	0.37474 (18)	0.33670 (9)	0.62856 (12)	0.0467 (3)	
C10	0.3070 (2)	0.39142 (9)	0.71775 (14)	0.0547 (4)	
C11	0.3689 (3)	0.49930 (12)	0.84739 (16)	0.0810 (6)	
H11A	0.2801	0.5356	0.8158	0.122*	
H11B	0.4670	0.5312	0.8762	0.122*	
H11C	0.3217	0.4679	0.9080	0.122*	
C12	0.24399 (19)	0.27684 (9)	0.57786 (13)	0.0533 (4)	
H12A	0.2757	0.2653	0.5007	0.064*	
H12B	0.1300	0.3034	0.5738	0.064*	
C13	0.22600 (17)	0.19546 (9)	0.63919 (12)	0.0441 (3)	
C14	0.1264 (2)	0.13340 (10)	0.58785 (13)	0.0585 (4)	
H14	0.0726	0.1426	0.5168	0.070*	
C15	0.1058 (2)	0.05841 (10)	0.64005 (15)	0.0628 (4)	
H15	0.0390	0.0175	0.6038	0.075*	
C16	0.1829 (2)	0.04364 (10)	0.74494 (14)	0.0578 (4)	
H16	0.1678	−0.0068	0.7806	0.069*	
C17	0.2824 (2)	0.10417 (10)	0.79654 (13)	0.0573 (4)	
H17	0.3355	0.0946	0.8677	0.069*	
C18	0.30491 (19)	0.17931 (9)	0.74421 (12)	0.0512 (4)	
H18	0.3741	0.2195	0.7802	0.061*	
O1	0.42556 (14)	0.44424 (7)	0.76084 (9)	0.0645 (3)	
O2	0.15923 (17)	0.38981 (8)	0.74826 (13)	0.0893 (5)	

### Atomic displacement parameters (Å^2^)

**Table:**

	*U*^11^	*U*^22^	*U*^33^	*U*^12^	*U*^13^	*U*^23^
C1	0.0861 (13)	0.0688 (12)	0.0800 (12)	−0.0192 (10)	0.0347 (10)	0.0019 (9)
C2	0.0454 (8)	0.0596 (9)	0.0473 (8)	−0.0022 (7)	0.0069 (6)	0.0007 (7)
C3	0.0513 (9)	0.0832 (13)	0.0529 (9)	−0.0022 (8)	0.0148 (7)	−0.0043 (8)
C4	0.0553 (10)	0.0832 (13)	0.0644 (10)	0.0093 (9)	0.0075 (8)	−0.0217 (9)
C5	0.0640 (10)	0.0587 (10)	0.0824 (12)	0.0037 (8)	0.0077 (9)	−0.0152 (9)
C6	0.0576 (9)	0.0523 (9)	0.0644 (10)	−0.0009 (7)	0.0137 (8)	−0.0010 (7)
C7	0.0424 (7)	0.0508 (8)	0.0437 (8)	0.0006 (6)	0.0050 (6)	0.0005 (6)
C8	0.0509 (8)	0.0461 (8)	0.0455 (8)	−0.0028 (6)	0.0093 (6)	0.0035 (6)
C9	0.0485 (8)	0.0449 (8)	0.0475 (8)	0.0012 (6)	0.0099 (6)	0.0085 (6)
C10	0.0562 (9)	0.0488 (9)	0.0607 (9)	0.0010 (7)	0.0203 (8)	0.0087 (7)
C11	0.0874 (13)	0.0835 (14)	0.0743 (12)	0.0042 (10)	0.0280 (10)	−0.0253 (10)
C12	0.0499 (9)	0.0599 (9)	0.0502 (8)	−0.0003 (7)	0.0028 (7)	0.0097 (7)
C13	0.0374 (7)	0.0516 (8)	0.0438 (7)	−0.0019 (6)	0.0083 (6)	0.0011 (6)
C14	0.0546 (9)	0.0716 (11)	0.0489 (8)	−0.0106 (8)	−0.0030 (7)	0.0000 (8)
C15	0.0607 (10)	0.0588 (10)	0.0692 (11)	−0.0163 (8)	0.0066 (8)	−0.0077 (8)
C16	0.0636 (10)	0.0472 (9)	0.0637 (10)	−0.0007 (7)	0.0172 (8)	0.0039 (8)
C17	0.0668 (10)	0.0565 (10)	0.0488 (9)	0.0022 (8)	0.0035 (7)	0.0073 (7)
C18	0.0555 (9)	0.0517 (9)	0.0461 (8)	−0.0068 (7)	−0.0011 (7)	−0.0004 (7)
O1	0.0613 (7)	0.0686 (7)	0.0651 (7)	0.0003 (6)	0.0204 (5)	−0.0162 (6)
O2	0.0663 (8)	0.0823 (9)	0.1234 (11)	−0.0109 (6)	0.0506 (8)	−0.0197 (8)

### Geometric parameters (Å, º)

**Table:**

C1—C2	1.500 (2)	C10—O1	1.3307 (19)
C1—H1A	0.9600	C11—O1	1.4368 (18)
C1—H1B	0.9600	C11—H11A	0.9600
C1—H1C	0.9600	C11—H11B	0.9600
C2—C3	1.389 (2)	C11—H11C	0.9600
C2—C7	1.4051 (19)	C12—C13	1.514 (2)
C3—C4	1.366 (2)	C12—H12A	0.9700
C3—H3	0.9300	C12—H12B	0.9700
C4—C5	1.370 (2)	C13—C18	1.380 (2)
C4—H4	0.9300	C13—C14	1.385 (2)
C5—C6	1.375 (2)	C14—C15	1.375 (2)
C5—H5	0.9300	C14—H14	0.9300
C6—C7	1.388 (2)	C15—C16	1.369 (2)
C6—H6	0.9300	C15—H15	0.9300
C7—C8	1.4701 (19)	C16—C17	1.368 (2)
C8—C9	1.3390 (18)	C16—H16	0.9300
C8—H8	0.9300	C17—C18	1.380 (2)
C9—C10	1.486 (2)	C17—H17	0.9300
C9—C12	1.498 (2)	C18—H18	0.9300
C10—O2	1.1981 (17)		
			
C2—C1—H1A	109.5	O1—C10—C9	113.84 (12)
C2—C1—H1B	109.5	O1—C11—H11A	109.5
H1A—C1—H1B	109.5	O1—C11—H11B	109.5
C2—C1—H1C	109.5	H11A—C11—H11B	109.5
H1A—C1—H1C	109.5	O1—C11—H11C	109.5
H1B—C1—H1C	109.5	H11A—C11—H11C	109.5
C3—C2—C7	118.37 (14)	H11B—C11—H11C	109.5
C3—C2—C1	120.06 (14)	C9—C12—C13	116.50 (12)
C7—C2—C1	121.57 (13)	C9—C12—H12A	108.2
C4—C3—C2	121.76 (15)	C13—C12—H12A	108.2
C4—C3—H3	119.1	C9—C12—H12B	108.2
C2—C3—H3	119.1	C13—C12—H12B	108.2
C3—C4—C5	119.96 (15)	H12A—C12—H12B	107.3
C3—C4—H4	120.0	C18—C13—C14	117.74 (13)
C5—C4—H4	120.0	C18—C13—C12	123.35 (13)
C4—C5—C6	119.70 (16)	C14—C13—C12	118.91 (13)
C4—C5—H5	120.1	C15—C14—C13	121.19 (15)
C6—C5—H5	120.1	C15—C14—H14	119.4
C5—C6—C7	121.39 (15)	C13—C14—H14	119.4
C5—C6—H6	119.3	C16—C15—C14	120.44 (15)
C7—C6—H6	119.3	C16—C15—H15	119.8
C6—C7—C2	118.77 (13)	C14—C15—H15	119.8
C6—C7—C8	121.87 (13)	C17—C16—C15	119.08 (15)
C2—C7—C8	119.34 (13)	C17—C16—H16	120.5
C9—C8—C7	128.28 (14)	C15—C16—H16	120.5
C9—C8—H8	115.9	C16—C17—C18	120.78 (14)
C7—C8—H8	115.9	C16—C17—H17	119.6
C8—C9—C10	119.27 (14)	C18—C17—H17	119.6
C8—C9—C12	125.57 (13)	C13—C18—C17	120.77 (14)
C10—C9—C12	115.11 (12)	C13—C18—H18	119.6
O2—C10—O1	122.14 (15)	C17—C18—H18	119.6
O2—C10—C9	124.02 (16)	C10—O1—C11	116.85 (12)
			
C7—C2—C3—C4	−1.9 (2)	C8—C9—C10—O1	−2.2 (2)
C1—C2—C3—C4	177.90 (16)	C12—C9—C10—O1	−179.66 (12)
C2—C3—C4—C5	0.2 (3)	C8—C9—C12—C13	96.20 (17)
C3—C4—C5—C6	1.0 (3)	C10—C9—C12—C13	−86.57 (16)
C4—C5—C6—C7	−0.3 (3)	C9—C12—C13—C18	8.8 (2)
C5—C6—C7—C2	−1.4 (2)	C9—C12—C13—C14	−170.83 (13)
C5—C6—C7—C8	−179.81 (15)	C18—C13—C14—C15	0.5 (2)
C3—C2—C7—C6	2.5 (2)	C12—C13—C14—C15	−179.90 (14)
C1—C2—C7—C6	−177.32 (16)	C13—C14—C15—C16	0.4 (2)
C3—C2—C7—C8	−179.11 (14)	C14—C15—C16—C17	−0.7 (2)
C1—C2—C7—C8	1.1 (2)	C15—C16—C17—C18	0.2 (2)
C6—C7—C8—C9	−39.7 (2)	C14—C13—C18—C17	−1.0 (2)
C2—C7—C8—C9	141.93 (15)	C12—C13—C18—C17	179.36 (13)
C7—C8—C9—C10	−179.57 (13)	C16—C17—C18—C13	0.7 (2)
C7—C8—C9—C12	−2.4 (2)	O2—C10—O1—C11	0.7 (2)
C8—C9—C10—O2	176.88 (16)	C9—C10—O1—C11	179.81 (14)
C12—C9—C10—O2	−0.5 (2)		

### Hydrogen-bond geometry (Å, º)

*Cg*1 is the centroid of the C13–C18 benzene ring.

**Table:**

*D*—H···*A*	*D*—H	H···*A*	*D*···*A*	*D*—H···*A*
C3—H3···*Cg*1^i^	0.93	2.87	3.7809 (17)	165

Symmetry code: (i) *x*+1, −*y*−1/2, *z*−3/2.
